# A Porphyrin Nanomaterial for Photoimmunotherapy for Treatment of Melanoma

**DOI:** 10.1002/advs.202414592

**Published:** 2025-04-09

**Authors:** Zhuang Fan, Qing Pei, Haojie Sun, Haiyan Zhang, Zhigang Xie, Tao Zhang, Chong Ma

**Affiliations:** ^1^ China‐Japan Union Hospital of Jilin University 126 Xiantai Street Changchun Jilin 130033 P. R. China; ^2^ State Key Laboratory of Polymer Physics and Chemistry Changchun Institute of Applied Chemistry Chinese Academy of Sciences Changchun Jilin 130022 P. R. China; ^3^ School of Applied Chemistry and Engineering University of Science and Technology of China Hefei Anhui 230026 P. R. China

**Keywords:** combination therapy, immunogenic cell death, melanoma, phototherapy, tumor vaccine

## Abstract

The incidence of melanoma, the third most common skin cancer, has been on the rise in recent years. In addition, it has a high mortality rate due to its high aggressiveness. Phototherapy, as a promising treatment method, can effectively kill tumor cells, but it is incapable of the treatment of tumor metastasis. Herein, a nanomaterial (TPC@OVA NPs) is developed for phototherapy in conjunction with immunotherapy against melanoma. TPC, as a derivative of porphyrin, is used as a photosensitizer with excellent biosafety and photostability. After assembly with ovalbumin (OVA), TPC@OVA NPs with vaccine properties is formed, which can not only ablate the primary tumor but also induce immunogenic cell death (ICD). In addition, DC cells can be stimulated to mature by exogenous OVA, enhancing the immune response against tumors by further activating T lymphocytes. Combined with immune checkpoint inhibitor aPD‐1, the immune microenvironment is reshaped, and the increased activity of immunotherapy are validated. This work highlights the potential of combining phototherapy and immunotherapy against metastasis.

## Graphical abstract

The TPC@OVA NPs are developed as dual‐functional nanomaterials combining phototherapy and immunotherapy for melanoma treatment. This system effectively ablates primary tumors, induces immunogenic cell death, promotes dendritic cell maturation, and enhances T cell activation. In combination with anti‐PD‐1 therapy, it remodels the tumor immune microenvironment and significantly enhances the efficacy of immunotherapy.

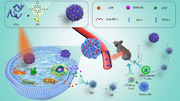

## 1 Introduction

Melanoma is the most aggressive type of skin cancer, and its incidence is steadily increasing.^[^ ^1^, ^2^ ^]^ There were ≈320000 new cases globally in 2020, with a mortality rate of 17.5%.^[^ ^3^ ^]^ Although scar resection, lymph node dissection, chemotherapy, radiotherapy, and immunotherapy showed some improvement in short‐term survival, no improvement in 5‐year survival was found.^[^ ^4^ ^]^ A new type of treatment is urgently needed to improve the treatment effects and increase the survival rate. Phototherapy depends on light and photosensitizers to generate heat or ROS to kill tumor cells,^[^ ^5^, ^6^, ^7^, ^8^ ^]^ mainly including photothermal and photodynamic therapy.^[^ ^9^, ^10^ ^]^ Phototherapy has the characteristics of high safety, strong controllability, and less trauma.^[^ ^11^, ^12^ ^]^ Although phototherapy can ablate the primary tumor to a large extent and inhibit the growth of the tumor,^[^ ^13^, ^14^ ^]^ the depth of laser penetration is always limited, and the killing effects of the deep tumor are not good.^[^ ^15^, ^16^, ^17^ ^]^ Due to the anoxic microenvironment of the tumor, insufficient ROS production is also a problem that cannot be ignored.^[^ ^18^, ^19^, ^20^ ^]^ In addition, phototherapy cannot effectively inhibit tumor metastasis because of the spatial and temporal limitations of light exposure.^[^ ^21^, ^22^ ^]^

For the past few years, immunotherapy has shown huge potential in cancer treatment,^[^ ^23^, ^24^ ^]^ especially for aggressive and metastatic tumors.^[^ ^25^ ^]^ Broadly speaking, tumor immunotherapy refers to a treatment method that produces tumor‐specific immune response through active or passive means and plays a role in inhibiting and killing tumor cells.^[^ ^26^ ^]^ Cancer immunotherapy mainly includes oncolytic virus therapy, cancer vaccine, cytokine therapy, adoptive cell transplantation, and immune checkpoint inhibitors.^[^ ^23^, ^27^, ^28^ ^]^ While single immunotherapy possesses a low tumor response and suboptimal antitumor effect. For example, aPD‐1 does not initiate immune system‐specific responses and target tumor cells, but only exerts its therapeutic function on its related pathways.^[^ ^29^ ^]^

Tumor vaccine, as a systemic anti‐tumor specific treatment, can induce T cells to differentiate into cytotoxic T cells through antigen presentation by DC cells, thus generating a durable anti‐tumor immune response.^[^ ^30^, ^31^, ^32^ ^]^ However, current cancer vaccines mainly suffer from low antigen loading rates.^[^ ^33^ ^]^ Although immunotherapy can induce a systemic anti‐tumor immune response, it is relatively ineffective in ablating solid primary tumors.^[^ ^34^ ^]^ Up to now, there has been a lot of work to combine phototherapy and immunotherapy for tumor therapy.^[^ ^35^, ^36^, ^37^, ^38^, ^39^ ^]^ Except for killing tumor cells, phototherapy can also cause ICD, release DAMPs and tumor‐associated antigens (TAEs), and stimulate DC cells to perform antigen presentation.^[^ ^40^, ^41^, ^42^, ^43^ ^]^ However, this immune response is often limited and insufficient to cause a comprehensive and lasting anti‐tumor effect.^[^ ^44^ ^]^ At this time, the combination of tumor vaccine and immune checkpoint blocking can effectively activate the immune system in the body, reverse the inhibitory immune microenvironment, remove the remaining tumor cells, and treat and prevent the distant metastasis of the tumor.^[^ ^45^, ^46^ ^]^

**Scheme 1:**
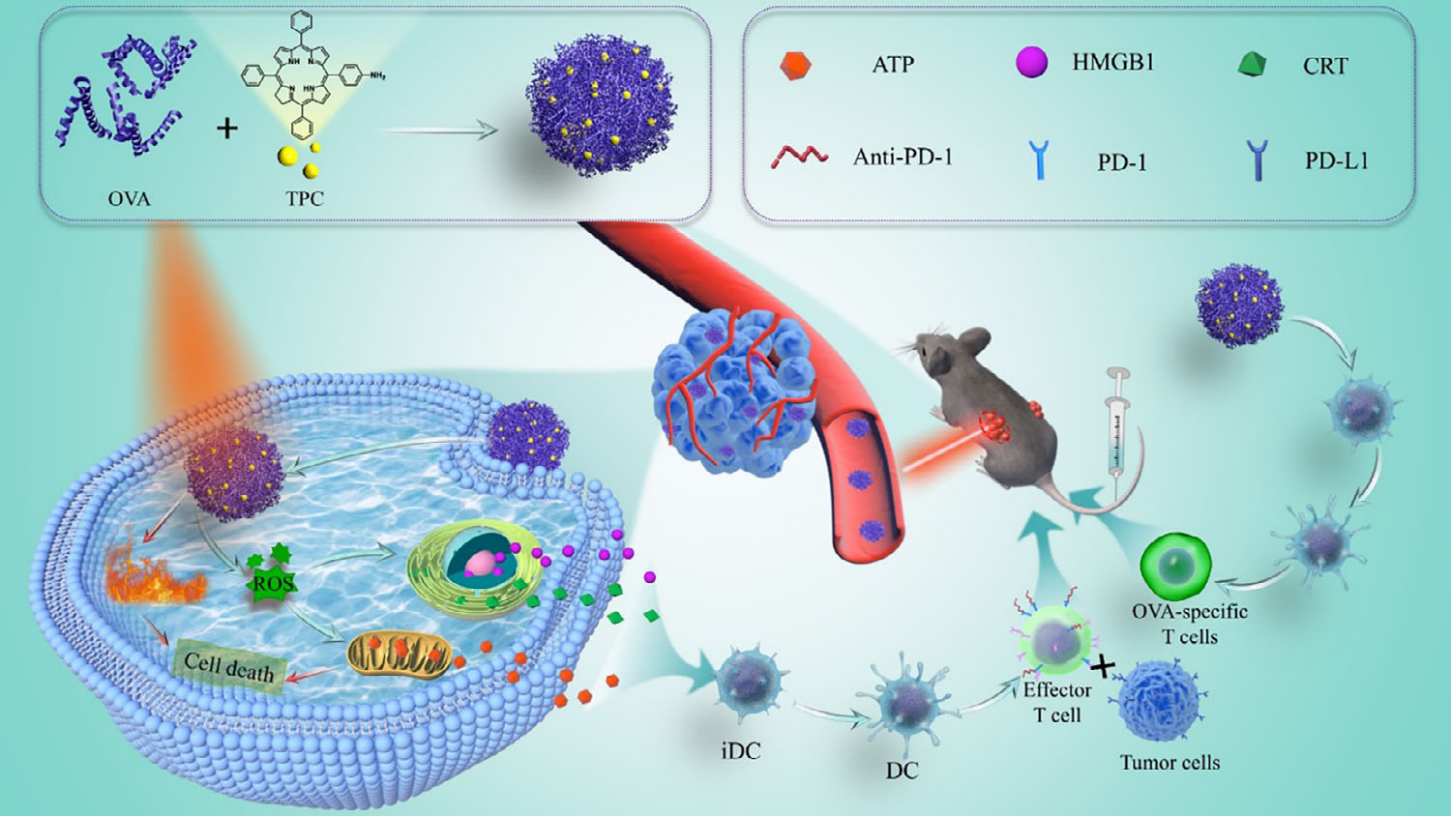
Schematic diagram of TPC@OVA NPs photoimmunotherapy for treatment of melanoma.

Here, a phototherapy combined immunotherapy by forming nano‐vaccine (TPC@OVA NPs) was developed. TPC is a kind of porphyrin material with outstanding photothermal and photodynamic properties, and OVA is an immune‐stimulating antigen. The nano‐vaccine is prepared by the assembly of TPC and OVA. This nano‐vaccine can effectively ablate the primary tumor and induce a systemic immune response (Scheme 1). After entering the cells and upon laser irradiation, TPC@OVA NPs generate heat and ROS to ablate the cells and induce ICD. Together with OVA, DC cells are stimulated to mature and the antigen is presented to T lymphocytes, inducing T lymphocyte activation. In addition, aPD‐1 blocks the PD‐1 receptor on cytotoxic T cells, preventing immune escape from tumor cells. This nano‐vaccine integrates multiple therapeutic modalities and effectively inhibits the growth of B16‐OVA tumors.

## 2 Results and Discussion

### 2.1 Characterization of TPC@OVA NPs

**Figure 1:**
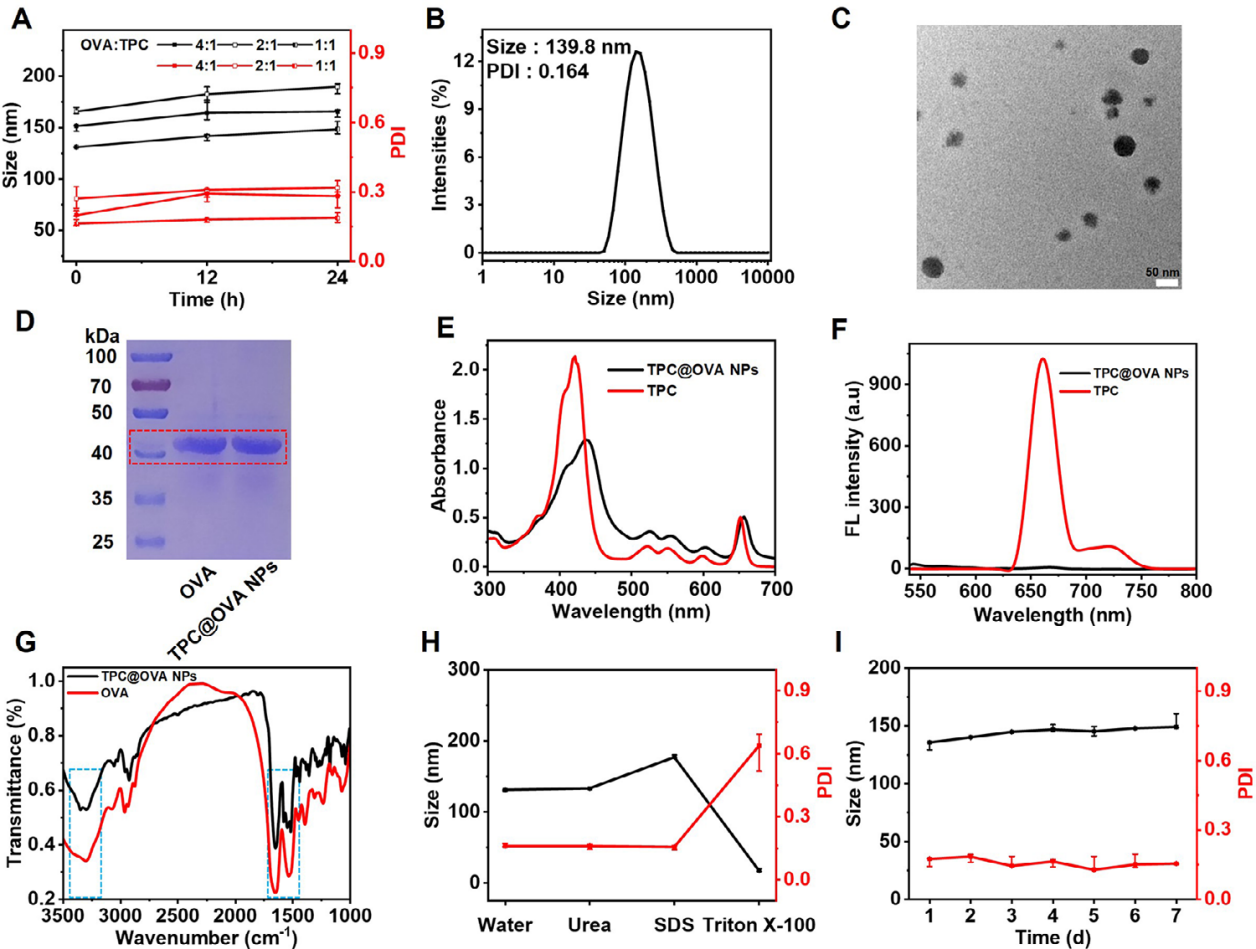
Characterization of TPC@OVA NPs. A) Particle size and PDI of TPC@OVA NPs with different proportions of OVA: TPC. B) Size of TPC@OVA NPs in deionized water. C) TEM image of TPC@OVA NPs. D) SDS‐PAGE analysis of OVA and TPC@OVA NPs. E) The UV–vis spectra of TPC and TPC@OVA NPs. F) The fluorescence spectra of TPC and TPC@OVA NPs. G) FTIR spectra of TPC@OVA NPs and OVA. H) Particle size and PDI change curves of TPC@OVA NPs after treatment with Urea, SDS, and Triton X‐100, respectively. I) Variations of hydrodynamic diameter and PDI of TPC@OVA NPs in PBS solution containing 10% FBS for a week. Bars express SD (n = 3).

The synthesis of TPC is based on previously reported work.^[^ ^47^ ^]^ TPC and OVA can assemble in water to form nanoparticles (TPC@OVA NPs) by the nanoprecipitation method. First, the optimal ratio of TPC to OVA was screened. When the ratio of OVA to TPC was 1:2, it was found that more precipitate was produced after stirring at this ratio. As shown in **Figure** **1**A, when the mass ratio of TPC to OVA is 1:1, TPC@OVA NPs have the smallest particle size. The ratio of TPC and OVA measured by UV is 1: 1.1 in TPC@OVA NPs. The particle size of TPC@OVA NPs has been measured by dynamic light scattering (DLS) to be ≈139.8 ± 3.7 nm (Figure 1B). As shown in Figure 1C, the regular spherical shape is seen under a transmission electron microscope (TEM). It is noteworthy that the Zeta potential of TPC@OVA NPs is −24.5±1.6 mV, and there is little change in Zeta potential after two weeks (Figure S1, Supporting Information).

As shown by gel electrophoresis of SDS‐PAGE, the two strips are at the same height, indicating that the TPC and OVA have been successfully joined together (Figure 1D). The UV–vis spectra of TPC@OVA NPs in deionized water and TPC in DMF were collected. Compared to TPC, the UV absorption spectrum of TPC@OVA NPs is somewhat redshifted and has enhanced absorbance at the Q‐band (Figure 1E). Further, the fluorescence of TPC and TPC@OVA NPs was measured. TPC@OVA NPs have quenched fluorescence, compared with TPC (Figure 1F), indicating the effective aggregation between TPC and OVA. Fourier transform infrared (FTIR) spectra of C═O (1651,1530 cm^−1^) and ─OH (3300 cm^−1^) indicates the existence of OVA in TPC@OVA NPs (Figure 1G). The presence of OVA in TPC@OVA NPs can also be validated from the CD spectral curve (Figure S2, Supporting Information).

To investigate the assembly mechanism of TPC@OVA NPs, the particle size of TPC@OVA NPs was measured after different treatments with urea, sodium dodecyl sulfate (SDS), and Triton X‐100 solutions. The result shows that the particle size of TPC@OVA NPs greatly reduces in Triton X‐100 solution while the polydisperse index (PDI) significantly rises (Figure 1H), confirming that hydrophobic interaction is the main driving force of assembly. In addition, it was investigated that the stability of TPC@OVA NPs in water, 5% glucose solution, and PBS solution including 10% fetal bovine serum for 7 days, respectively. The variations of particle size and PDI of TPC@OVA NPs are ignorable (Figure 1I; Figures S3 and S4, Supporting Information). As is exhibited in Figure S5 (Supporting Information), the release of OVA in a weak acid environment is much faster than that in PBS (pH = 7.4).

### 2.2 Photothermal and Photodynamic Property of TPC@OVA NPs in Vitro

**Figure 2:**
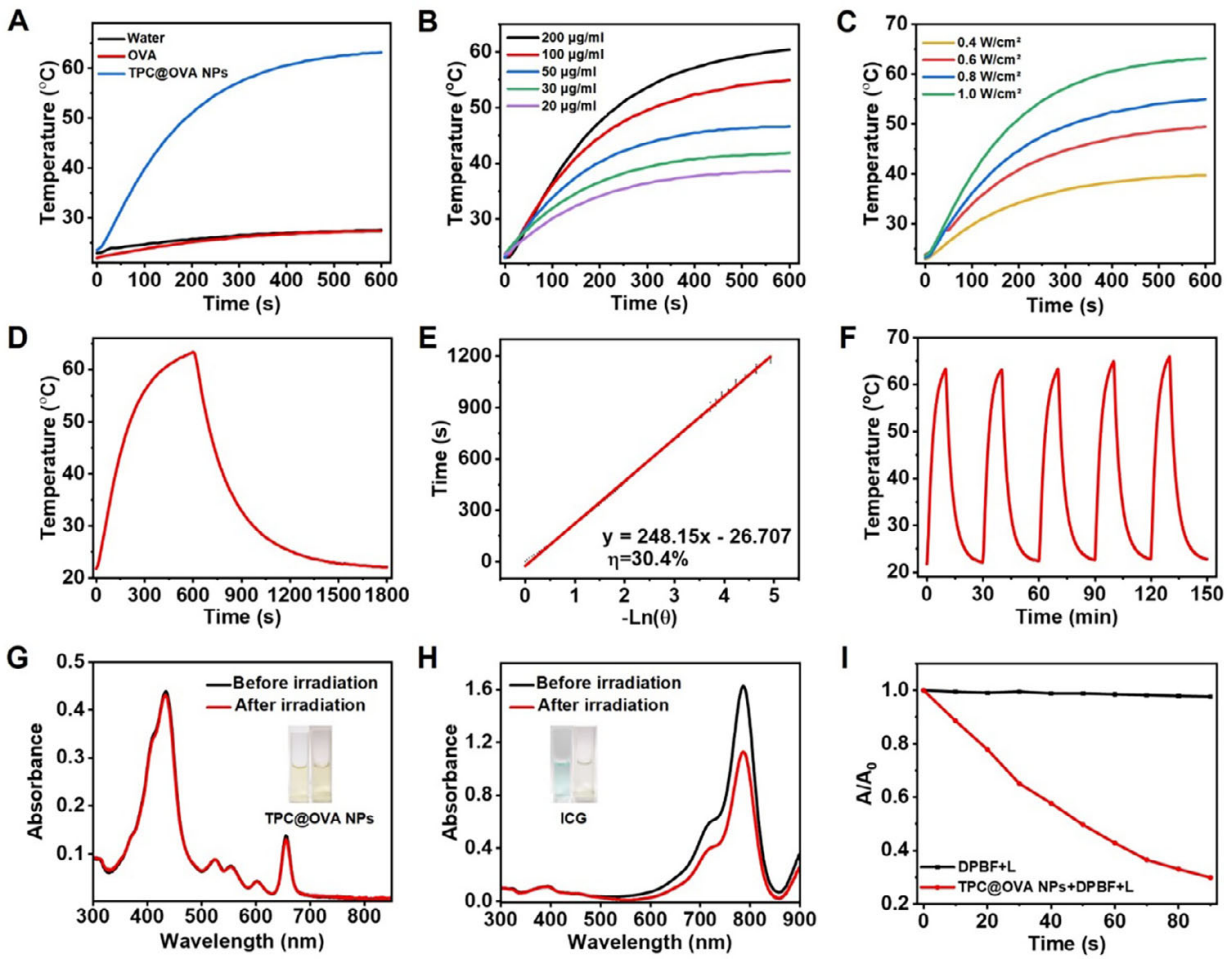
Photothermal and photodynamic performance of TPC@OVA NPs. A) The temperature changes of water, OVA, and TPC@OVA NPs under illumination. B) Warming curves of different concentrations of TPC@OVA NPs after illumination. C) Temperature rise curves of TPC@OVA NPs irradiated at different power densities. D) Heat‐up and cool‐down curves of TPC@OVA NPs. E) A linear relationship between negative logarithm of temperature change and cooling time. F) Temperature changes of TPC@OVA NPs during 5 cycles of heating and cooling. Changes in UV absorption values of TPC@OVA NPs G) and ICG H) before and after illumination (inset: before illumination on the left and after illumination on the right). I) UV absorption curves at 417 nm of DPBF after different treatments (L:685 nm, 0.2 W cm^−2^).

In the case of equal laser irradiation, the TPC@OVA NPs with a mass ratio of 1:1 for TPC to OVA have the best photothermal performance (Figure S6, Supporting Information). The temperature of the TPC@OVA NPs solution rises by 40 °C in 10 min of exposure to a 685 nm laser with 1.0 W cm^−2^ power (**Figure** **2**A). The temperature of the OVA solution and water only slightly increases under the same condition, suggesting that the heat is generated by TPC@OVA NPs. The temperature fluctuation is dependent on the concentration of TPC@OVA NPs (Figure 2B). In addition, the temperature rises with the increasing laser power (Figure 2C). The photothermal conversion efficiency is determined by tracking the temperature change curve from heating to cooling to room temperature (Figure 2D). The photothermal conversion efficiency is ≈30.4% (Figure 2E).

To examine the photothermal stability of TPC@OVA NPs, we recorded temperature changes over five light cycles and discovered that the temperature increase was nearly constant (Figure 2F). After 5 light cycles, the average particle size of TPC@OVA NPs slightly changed, and the nanostructure of TPC@OVA NPs was not damaged (Figures S7 and S8, Supporting Information). Additionally, the nanoparticle's photostability was investigated. After 10 min of laser illumination, the absorbance of TPC@OVA NPs did not appreciably alter (Figure 2G). While the absorption value of indocyanine greens drastically dropped (Figure 2H). Moreover, the illustration exhibited the remarkable photostability of TPC@OVA NPs without color change. As shown in Figure S9 (Supporting Information), the CD signal of TPC@OVA NPs changes only slightly, proving that OVA can remain stable after irradiation. The capacity of TPC@OVA NPs to generate reactive oxygen species was examined by using DPBF. The UV absorption value of DPBF steadily dropped at 417 nm with an increase in illumination time, however, that in control groups remained relatively constant ((Figure 2I; Figures S10 and S11, Supporting Information). This result implies the effective generation of singlet oxygen by TPC@OVA NPs.

### 2.3 Cellular Uptake and Cytotoxicity of TPC@OVA NPs

**Figure 3:**
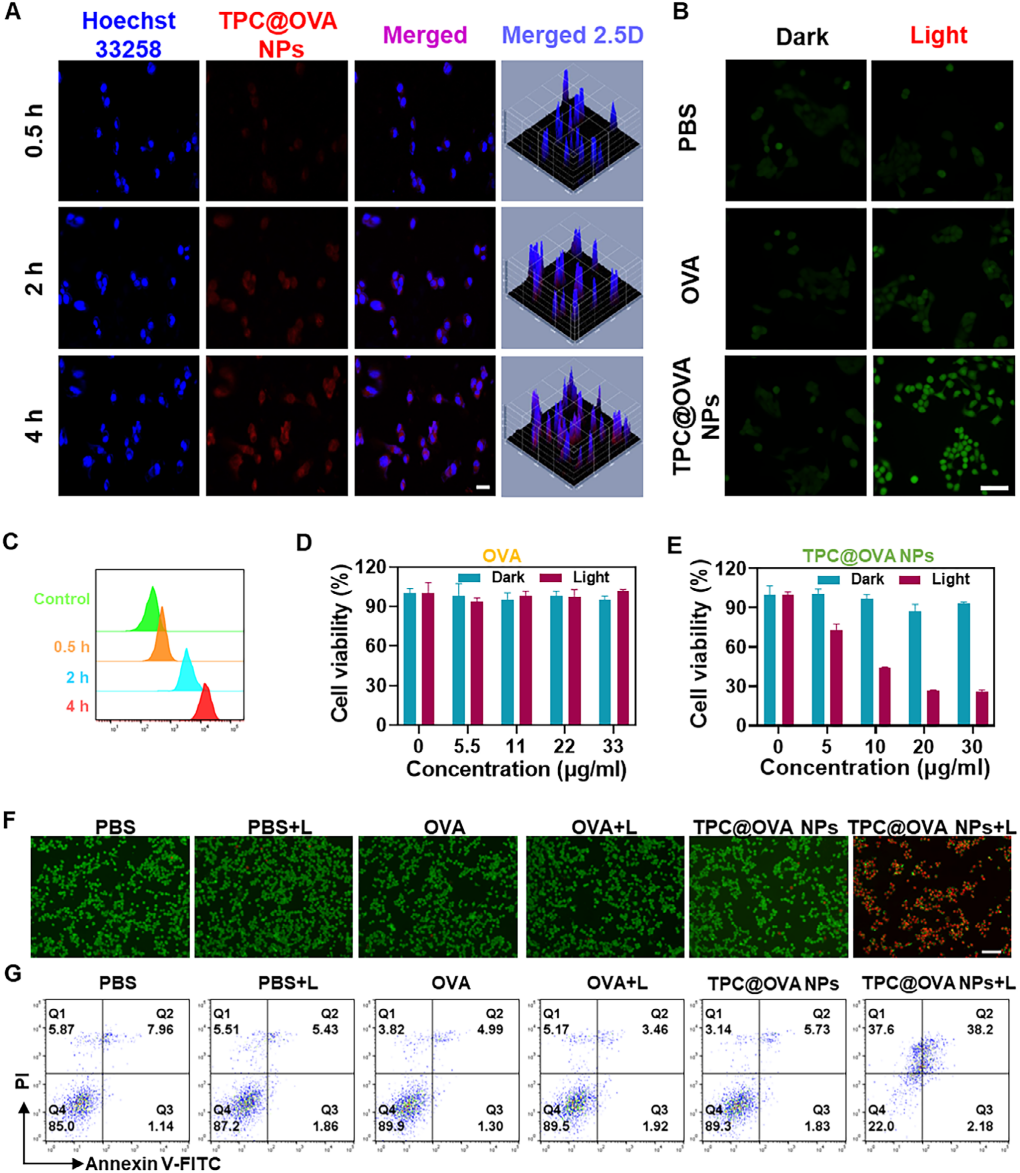
Phototherapy performance of TPC@OVA NPs in cells. A) Fluorescent pictures of B16‐OVA cells treated with TPC@OVA NPs for 0.5, 2, and 4 h. Scale bar, 20 µm. B) The fluorescence of ROS in cells after different treatments. Scale bar, 50 µm. C) Quantitative fluorescence of B16‐OVA cells after incubation with TPC@OVA NPs for 0.5, 2, and 4 h by FCM. Cytoactive of B16‐OVA cells treated with OVA D) and TPC@OVA NPs E). (F) Fluorescence images of live and dead cells co‐stained B16‐OVA cells treated with different conditions. Scale bar, 100 µm. G) The analysis of apoptotic and necrotic B16‐OVA cells after various treatments. L: 685 nm, 0.6 W  cm^−2^. Bars express SD (n = 3).

B16‐OVA cells were incubated with TPC@OVA NPs at 37 °C. The cells' red fluorescence gradually enhanced as the incubation period increased (**Figure** **3**A), and a similar time‐dependent endocytosis was validated by flow cytometry (Figure 3C; Figure S12, Supporting Information). These findings suggest that TPC@OVA NPs can be efficiently absorbed by B16‐OVA cells. The same results were obtained by using DC2.4 cells (Figure S13, Supporting Information). The intracellular ROS generation was detected by DCFH‐DA probes. The cells in the TPC@OVA NPs+L group had intense green fluorescence (Figure 3B), compared with that in control groups. The cytotoxicity of TPC@OVA NPs was measured by 3‐(4,5‐dimethylthiazol‐2‐yl)‐2,5‐diphenyltetrazolium bromide (MTT) assays. The OVA (33 µg mL^−1^) had hardly any effect on cellular activity whether there is light irradiation or not (Figure 3D). As shown in Figure 3E, neither laser irradiation (685 nm, 0.6 W cm^−2^) nor TPC@OVA NPs in dark conditions can cause cellular death. However, the cell viability was dropped with the increase of TPC@OVA NPs concentration upon irradiation. The TPC@OVA NPs were incubated with DC2.4 cells, and the cell viability was above 90% (Figure S14, Supporting Information). Additionally, cells were stained with propyl iodide (PI) and calcein acetoxy‐methyl ester (calcein‐AM) after various treatments. It was found that only the TPC@OVA NPs+L group exhibited intense red fluorescence (Figure 3F). Apoptotic cells were also quantified by flow cytometry. Compared to the other groups, the TPC@OVA NPs+L group had a significantly larger quantity of apoptotic cells (Figure 3G). These results indicate that TPC@OVA NPs effectively kill tumor cells under laser irradiation.

### 2.4 Immunoactivities Capacity of TPC@OVA NPs in Vitro

**Figure 4:**
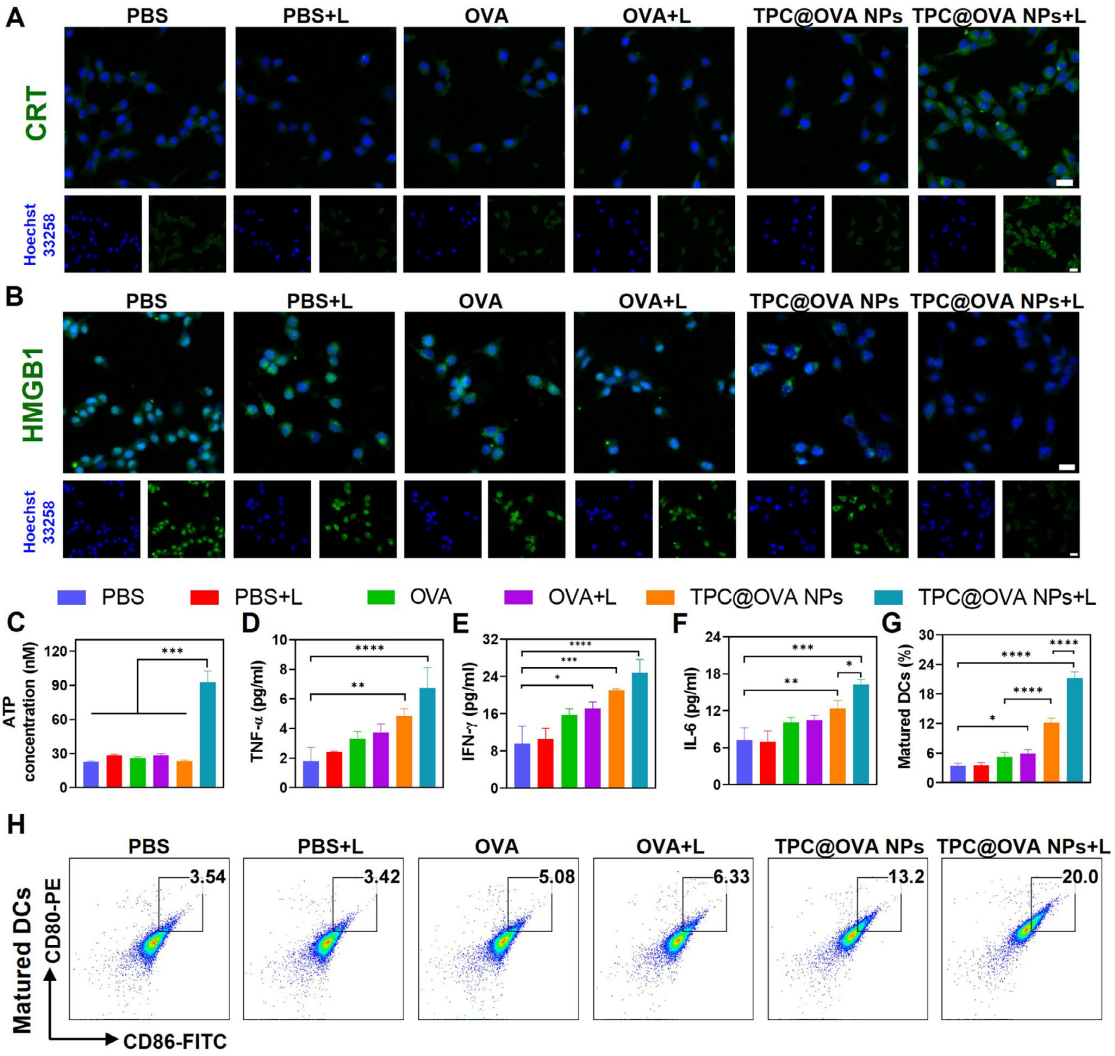
In vitro immune response. CLSM images of CRT A) and HMGB1 B) exposure in B16‐OVA cells after various treatments. Scale bar, 20 µm. C) The content of ATP in each group supernatant. Immuno‐inflammatory cytokines secretion of TNF‐α D), IFN‐γ E), and IL‐6 F) in different groups detected by ELISA. G) The quantification of DC2.4 cells maturation after different treatments. H) Analysis of DC2.4 cells maturation by FCM after incubation with different cellular supernatants. Means ± SD (n = 3). *p < 0.05, **p < 0.01 and ***p < 0.001 and ****p <0.0001.

The immunogenic cell death (ICD) is often accompanied by the release of damage‐associated molecular patterns (DAMPs) which mainly include calretin (CRT), ATP, and high mobility histone B1 (HMGB1). DAMPs act as immunogenic signals to stimulate the maturation of DC cells and activate T lymphocytes, thus triggering an anti‐tumor immune response.^[^ ^48^, ^49^ ^]^ The expression and release of DAMPs in B16‐OVA cells after various treatments were verified by immunofluorescence. The green fluorescence of cells treated by TPC@OVA NPs+L was substantially more intensive than those in other groups, indicating that endoplasmic reticulum stress occurred in B16‐OVA cells, and CRT everted onto the cell membrane (**Figure** **4**A). For the HMGB1, the TPC@OVA NPs+L treatment led to less green fluorescence than that of the other groups, indicating that HMGB1 in the nucleus is released into the extracellular space (Figure 4B). Then, the ATP content in the treated cell supernatants was measured, and the results indicate that the treatment of TPC@OVA NPs+L secreted ≈3 times more ATP than other groups (Figure 4C).

DC cells are professional antigen presenting cells, so the maturation of DC cells is the key to the success of immunotherapy.^[^ ^50^, ^51^ ^]^ Here, we investigated the effects of B16‐OVA cells with different treatments on DC cell maturation. The expression of CD80/CD86 was detected by flow cytometry. As is exhibited in Figure 4H and G, PBS, PBS+L, OVA, OVA+L, TPC@OVA NPs, TPC@OVA NPs+L respectively induced 3.54%, 3.42%,5.08%, 6.33%, 13.2%, 20.0% CD80^+^CD86^+^ DCs. As a model antigen, OVA can be internalized by APCs and achieve antigen presentation, thus promoting the maturation of DC cells. The above results suggest that the delivery system could increase the internalization of OVA in cells. Next, by co‐incubating various B16‐OVA cell supernatants with DC cells, TNF‐α, IFN‐γ, and IL‐6 in DC cells were determined by ELISA kit. The results showed that the content of these proteins in the TPC@OVA NPs+L group was the highest (Figure 4D–F). In summary, these results prove that TPC@OVA NPs can promote the expression and release of DAMPs, further stimulate the maturation of DC cells, and enhance the immune response.

### 2.5 Anti‐Tumor Ability and Biosafety of TPC@OVA NPs in Vivo

**Figure 5:**
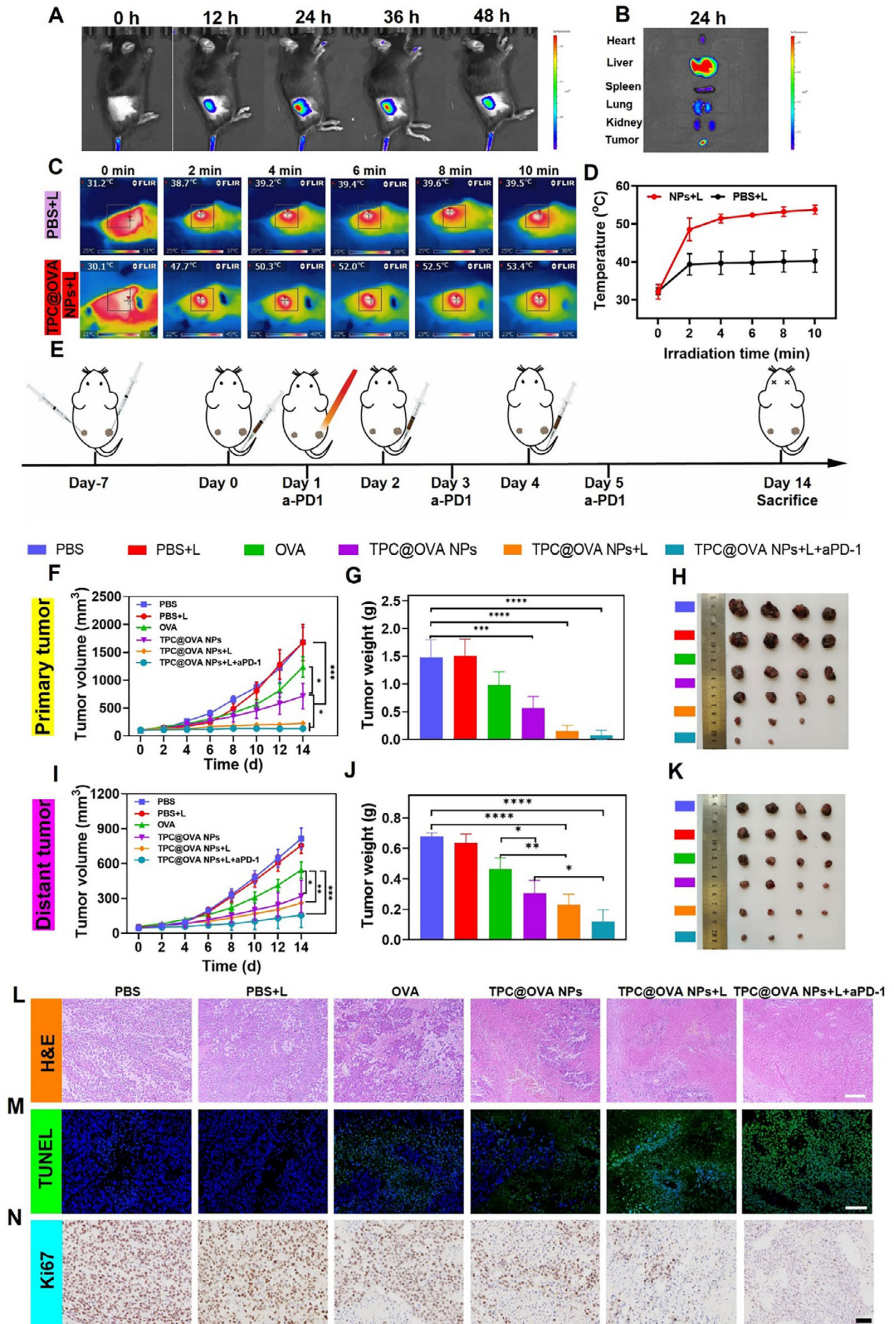
The antitumor effect in vivo. A) In vivo fluorescence images of tumors after intravenous injection of TPC@OVA NPs for different times. B) Fluorescent images of major organs 24 h after injection. C) Infrared thermography of tumor sites during irradiation (685 nm laser, 0.6 W cm^−2^) in mice 24 h after intravenous injection with TPC@OVA NPs (6 mg kg^−1^). D) Temperature detection values of tumor sites in mice during 10 min of illumination. E) The timeline of the mice receiving various treatments. Volume of in situ F) and distal I) tumors in each group of mice. The weights G) and photos H) of primary tumors in different groups. The weights J) and photos K) of distant tumors in each group. H&E L) and TUNEL M) staining of excised tumor sections of each group. Scale bar: 100 µm. N) Ki67 staining of slices of excised tumors after various treatments. Scale bar: 50 µm.*P < 0.05, **P < 0.01, and ***P < 0.001, ****p <0.0001.

In order to evaluate the anti‐tumor effect of TPC@OVA NPs, a bilateral B16‐OVA tumor model was established in C57BL/6 mice. In order to investigate the maximum accumulation time in the tumor, in vivo imaging was performed after TPC@OVA NPs (9 mg mL^−1^) were injected from the tail vein. The images indicated that the fluorescence at the tumor site was strongest at 24 h (**Figure** **5**A; Figure S15, Supporting Information). The ex vivo imaging of the organs at 24 h revealed the main accumulation of TPC@OVA NPs in the tumor and liver (Figure 5B). The mice were randomly divided into 6 groups with different treatments of PBS, PBS+L, OVA, TPC@OVA NPs, TPC@OVA NPs+L, TPC@OVA NPs+L+ aPD‐1 through the caudal vein at day 0, 2, and 4. In addition, aPD‐1 was injected into mice via intraperitoneal injection and only the primary tumor was illuminated with a 685 nm laser 1 day after TPC@OVA NPs administration (Figure 5E). After 10 min of radiation, the temperature at the tumor site in the TPC@OVA NPs+L group rose to 53.4 °C, while that in the PBS+L group only reached 39.5 °C (Figure 5C,D).

Compared with the PBS group, OVA treatment had a slight suppression effect on the primary tumor, and TPC@OVA NPs were superior to OVA for tumor treatment. TPC@OVA NPs+L and TPC@OVA NPs+L+ aPD‐1 treatments showed a notable inhibition in tumor growth, with nearly complete ablation of the primary tumor (Figure 5F–H). It is worth noting that TPC@OVA NPs+L+aPD‐1 showed a significantly stronger therapeutic effect on distant tumors than other controls (Figure 5I–K). H&E stained sections of in vitro tumors showed that nuclear ablation in the TPC@OVA NPs+L+ aPD‐1 group was the most abundant (Figure 5L). Subsequently, TDT‐mediated dUTP Notch end labeling (TUNEL) assay in primary tumors showed the most obvious apoptosis in TPC@OVA NPs+L+ aPD‐1 group (Figure 5M). In addition, Ki67 immunohistochemical results of in vitro tumors showed that cell proliferation was the least in the TPC@OVA NPs+L+ aPD‐1 group (Figure 5N). Then, the biosafety of TPC@OVA NPs was assessed by recording the mice's weight changes throughout the course of treatment. The weight of the mice of different groups continued to increase (Figure S16, Supporting Information). In addition, blood routine and blood biochemical tests of mice in all groups were normal (Figures S17 and S18, Supporting Information), and no abnormalities were detected in H&E sections of isolated important organs (Figure S19, Supporting Information).

### 2.6 Immune Response Capacity of TPC@OVA NPs in Vivo

**Figure 6:**
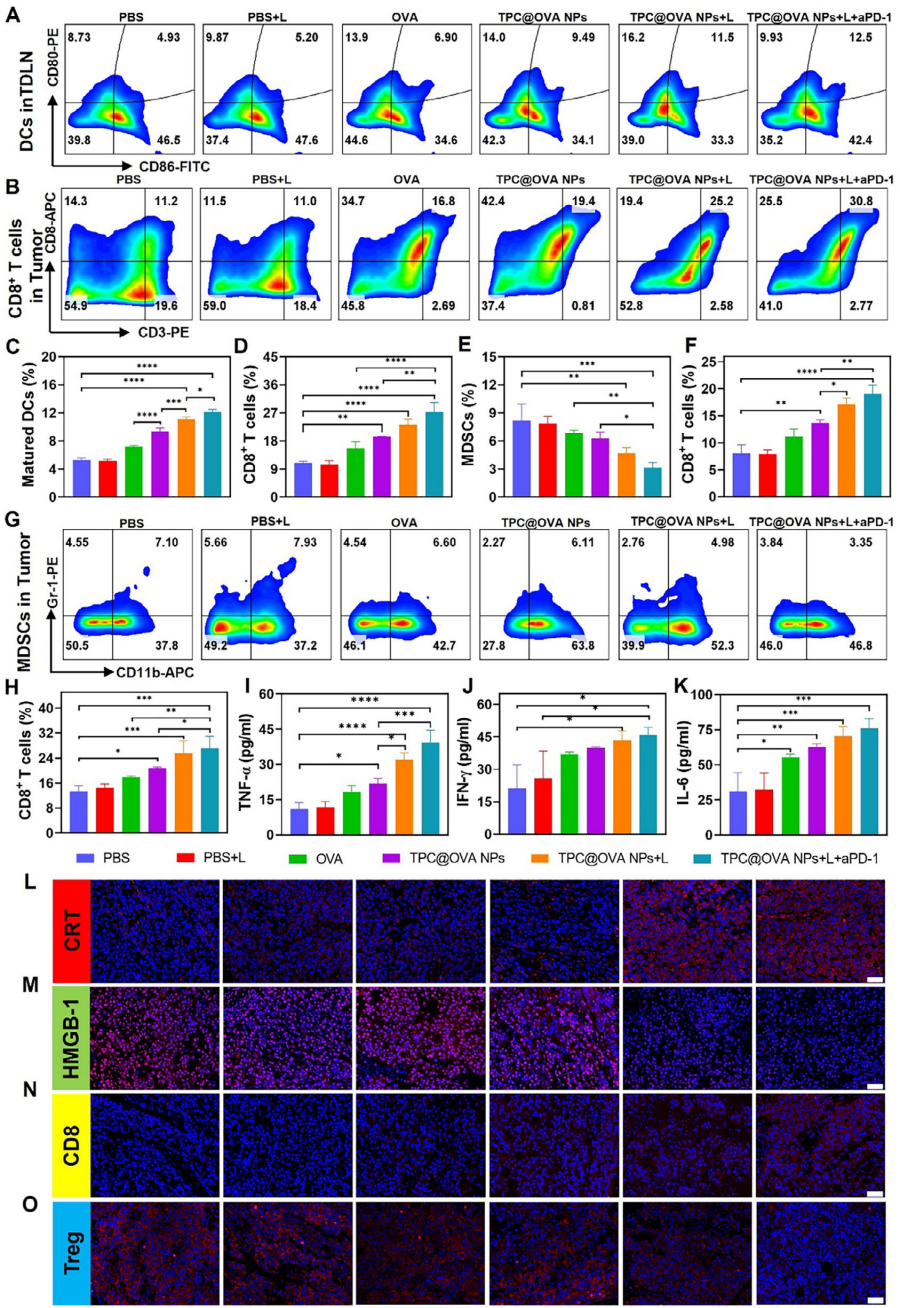
In vivo immunostimulatory ability. FCM analysis and the corresponding quantitative result of maturated DCs A, C) in TDLN in different groups. FCM analysis and the corresponding quantitative result of CD8^+^ T cells B, D) and MDSCs E, G) in tumors of different groups. Corresponding quantitative analysis of CD8^+^ T cells within spleen F) and TDLN H) in different groups. Cytokine levels of TNF‐α I), IFN‐γ J), and IL‐6 K) after different treatments. Immunofluorescence staining results of CRT L), HMGB‐1 M), CD8 T cells N), and Treg cells O) expression in tumor tissues of different groups. Scale bar: 50 µm. *P < 0.05, **P < 0.01, and ***P < 0.001, ****p <0.0001.

The immune response in mice was evaluated by examining the infiltration of immune cells in the lymph nodes, spleen, and tumors. First, the maturation of DC cells was analyzed by flow cytometry. In the inguinal lymph nodes, the DCs maturation with the treatment of TPC@OVA NPs, TPC@OVA NPs+L, and TPC@OVA NPs+L+ aPD‐1 was increased, and the TPC@OVA NPs+L+ aPD‐1 facilitated the maturation of DC cells most significantly (**Figure** **6**A and C). The same results were seen in the spleen (Figures S20 and S21, Supporting Information). After DC cells were stimulated, CD80 and CD86 molecules on the cell surface were overexpressed, which further activated T lymphocytes. As shown in Figure 6B and D, the activation of CD8 T lymphocytes in the TPC@OVA NPs+L+ aPD‐1 group was about three times that of the PBS group. For the lymph nodes and spleen, the activation of CD8 T lymphocytes in the TPC@OVA NPs+L+ aPD‐1 group was about twice that of the PBS group (Figure 6F, H; Figures S22 and S23, Supporting Information). It can be seen from CD8 immunofluorescence sections of the tumor in vitro that TPC@OVA NPs+L+ aPD‐1 group has obvious red fluorescence (Figure 6N). T‐lymphocytes are activated and differentiated into cytotoxic T‐lymphocytes, which exert anti‐tumor effects. These results validate that TPC@OVA NPs+L+ aPD‐1 has excellent anti‐tumor immune response ability.

In addition, the contents of TNF‐α, IFN‐γ, and IL‐6 in the blood of mice were detected. Compared with the PBS group, the expression level in the TPC@OVA NPs+L+ aPD‐1 group was significantly increased (Figure 6I–K). Then, immunofluorescence sections of tumor tissues show that TPC@OVA NPs+L+ aPD‐1 treatment resulted in obvious CRT expression and HMGB‐1 release, confirming the generation of ICD in vivo (Figure 6L,M). Then, changes in the immunosuppressive microenvironment were examined. MDSCs and Tregs inhibit the activation of CD8 T cells, thereby inducing immune escape. As is demonstrated in Figure 6E,G, the expression of MDSCs in the TPC@OVA NPs+L+ aPD‐1 group was significantly reduced compared with the PBS group. It was also found from immunofluorescence sections of isolated tumors that the TPC@OVA NPs+L+ aPD‐1 group showed the least red fluorescence compared with other groups (Figure 6O). These results demonstrate that TPC@OVA NPs+L combined with aPD‐1 significantly activate the anti‐tumor immune response and improve the immune microenvironment in vivo.

## 3 Conclusion

In summary, we designed a photoactive nanomaterial (TPC@OVA NPs) for phototherapy in conjunction with immunotherapy for melanoma. The nanomaterial, which is composed of porphyrin derivative (TPC) and chicken egg albumin (OVA), not only has excellent photothermal and photodynamic properties but also has good colloidal and photostability. TPC@OVA NPs can effectively accumulate in tumors and rapidly generate heat and ROS upon irradiation. While killing tumor cells at the primary site, TPC@OVA NPs can also cause ICD, cooperate with exogenous OVA to stimulate the maturation of DC cells, complete antigen presentation, and stimulate the differentiation of T cells. Then combined with immune checkpoint inhibitor aPD‐1, the immune microenvironment was reshaped to achieve systemic anti‐tumor immune response. Therefore, this study provides a new therapeutic strategy against melanoma.

## 4 Experimental Section

### Materials

3‐(4,5‐dimethylthiazol‐2‐yl)‐2,5‐diphenyltetrazolium bromide (MTT) and ovalbumin were purchased from Shanghai yuanye Bio‐Technology Co., Ltd. Anti‐HMGB1 antibody, anti‐Calreticulin (CRT), Donkey Anti‐rabbit IgG/FITC, anti‐Mouse CD86‐FITC, Gr‐1‐PE, CD80‐PE, CD11c‐APC, CD8a‐APC, CD11b‐APC, CD3ε‐FITC, BCA Protein Assay kit and Mouse TNF‐α Elisa Kit and IFN‐γ were purchased from Proteintech Co, Ltd Enhanced ATP Assay Kit was purchased from Beyotime Co, Ltd.

### Preparation of TPC@OVA NPs

The acetone solution of TPC (2 mg mL^−1^, 0.5 mL) was added to the OVA water solution (0.2 mg mL^−1^, 5 mL) in drops. Then, the mixture was stirred for 24 h, and the supernatant was removed by centrifugation at 3500 r min^−1^ for 5 min. Finally, the supernatant was added to the ultrafiltration tube (100 KDa) and centrifuged for 3500 r min^−1^ for 5 min to remove free OVA.

### Cell Culture

Aspirate the medium from the dish, add PBS, and wash 1–2 times, add 2 mL trypsin, and place in the incubator for digestion (B16‐OVA cells digested for 90 s, DC2.4 cells digested for 60 s). Add 2 mL of medium to plant digestion, blow down the cells completely, to the centrifuge tube centrifugation (1000 r min^−1^, 5 min), discard the supernatant, add 2 mL of medium to resuspend, take part of the resuspension solution to add to the petri dish containing 8 mL of fresh medium.

### Statistical Analysis

Data were expressed as mean ± standard deviation (SD). All experimental data in this experiment were statistically analyzed using GraphPad. Prism. 9.0.

## Conflict of Interest

The authors declare no conflict of interest.

## Supporting information



Supporting Information

## Data Availability

The data that support the findings of this study are available from the corresponding author upon reasonable request.
