# Supplementary material for: A Porphyrin Nanomaterial for Photoimmunotherapy for Treatment of Melanoma
Source: Adv Sci (Weinh). 2025 Apr 9;12(21):2414592. doi: 10.1002/advs.202414592 (PMC12140306; doi:10.1002/advs.202414592)
Supplement: Supplementary file 1 — Supporting Information [file ADVS-12-2414592-s001.docx]

**Supporting information**

**A Porphyrin Nanomaterial for Photoimmunotherapy for Treatment of Melanoma**

*Zhuang Fan, Qing Pei, Haojie Sun, Haiyan Zhang, Zhigang Xie^*^, Tao Zhang^*^, Chong Ma^*^*

**Experimental section**

**1. Materials**

Cell viability (live-dead cell staining) assay kit, Annexin V-FITC/propidium iodide (PI) double staining cell apoptosis detection kit and Hoechst 33258 were purchased from Jiangsu KeyGEN Biotechnology. Confocal laser scanning microscopy (CLSM) images were taken using a Zeiss LSM 700 (Zurich, Switzerland). The morphology of the nanoparticles (NPs) was measured by transmission electron microscopy (HT-7700). The size and the size distribution of nanoparticles were determined by dynamic light scattering (DLS) using a Malvern Zeta-sizer Nano. Absorption and Fluorescence spectra were recorded on Shimadzu UV-2450 and PerkinElmer LS-55 spectra fluorophotometer, respectively. The flow cytometry measurement was performed on a Becton Dickinson FACS Aria sorting flow cytometer (Becton-Dickinson, USA).

**2. Methods**

**2.1 Synthesis of TPC.**

TPC is synthesized according to the previous work.^[1]^

**2.2 Photothermal properties of TPC@OVA NPs**.

The TPC@OVA NPs, water and OVA solution were irradiated with a 685 nm laser at intensity of 0.6 W cm^−2^ for 600 s. TPC@OVA NPs in solution with various concentrations (20, 30, 50, 100, 200 µg mL^−1^) were irradiated with 685 nm laser at a power density of 0.8 W cm^−2^ for 600 s. Then TPC@OVA NPs (200 µg mL^−1^) were irradiated with laser at different power densities (0.4, 0.6, 0.8 and 1.0 W cm^-2^). The photothermal response of TPC@OVA NPs was recorded under laser irradiation for 10 min, and then the laser was turned off. To evaluate the photothermal stability, the temperature changes of TPC@OVA NPs (laser power: 1.0 W cm^−2^, 600 s, 200 μg mL^-1^) in aqueous solution were measured over 5 cycles of heating & cooling. The data above were recorded every 10 s by a thermocouple.

**2.3 Photothermal conversion efficiency.**

The photothermal conversion efficiency (𝜂) was calculated according to the equation (Eq) as follows according to published methods.

^RS1^ $\eta=\frac{hA(T_{Max}-T_{surr}) ‒ Q_{dis}}{I (1 ‒ {10}^{-A 685})}$, (1)

where h and A respectively represent the heat transfer coefficient and the surface area of the container, Tmax and Tsurr represent the maximum temperature and the room temperature of the environment, Q_dis_ represents the heat dissipation of the solvent (water), I is the laser power employed, and A_685_ is the absorbance of TPC@OVA NPs at 685 nm. The value of hA is calculated from the following equation

$\tau_{s}=\frac{m_{D}c_{D}}{hA}$ , (2)

where τs is the time constant for heat transfer of the system, which was determined from Figure 2E, m_D_ and c_D_ are the mass and heat capacity, respectively, of the deionized water used to disperse the NPs. Q_dis_ represents the heat dissipation from the laser absorbed by the water, so Q_dis_ was calculated according to the following equation

$Q_{dis} =\frac{c_{D}m_{D}\left( T_{Max(water)}-T_{surr}) \right.}{\tau_{s(water)}}$ , (3)

where Tmax(water) is the highest temperature of water, thus, Q_dis_ can be calculated. According to the obtained data and Equation (1), the photothermal conversion efficiency of the TPC@OVA NPs was determined.

**2.4 Intracellular detection of ROS.**

As a fluorescent ROS probe, 2′,7′-dichlorofluorescein diacetate (DCFH-DA) was used to detect ROS generation in cells. First, B16-OVA cells were treated with OVA and TPC@OVA NPs for 4 h, and then irradiated with or without 685 nm laser (0.6 W cm^-2^) for 5 min followed by washing with phosphate-buffered saline (PBS) for 3 times. Subsequently, DCFH-DA was added, and cells were incubated for 20 min. After the media were removed, the cells were washed with PBS and observed by the Operetta CLS^TM^ High Content Analysis System (PerkinElmer, USA).

**2.5 Cell viability assays**.

The cytotoxicity of OVA and TPC@OVA NPs with or without laser irradiation was investigated by the classical 3-(4,5-dimethylthiazol-2-yl)-2,5-diphenyltetrazolium bromide (MTT) assay. B16-OVA cells or DC2.4 cells at a density of 10^4^ cells per well were inoculated into 96-well culture plates, and cultured for 24 h. Then TPC@OVA NPs (0-30 μg mL^-1^) were added, and the cells were incubated for 4 h followed by irradiation with a 685 nm laser (0.6 W cm^-2^) for 5 min for light groups. After incubation at 37 ^o^C for 20 h, 20 μL of MTT solution (5 mg mL^-1^) was added to each well. After 4 h, the media were removed and 150 μL of dimethyl sulfoxide (DMSO) was added into each well to dissolve the formazan crystals. Finally, the absorbance of each well at 490 nm was measured by a microplate reader.

**2.6 Live-dead cell staining.**

B16-OVA cells were pretreated with PBS, OVA or TPC@OVA NPs (30 μg mL^-1^), and 4 h later, the cells were irradiated with a 685 nm laser (0.6 W cm^-2^) for 5 min for light groups. After 20 h of incubation at 37 ^o^C, cells were stained with calcein-AM/propidium iodide (PI) solution for 30 min at room temperature. Finally, the samples were imaged by a fluorescence microscope.

**2.7 Cell apoptosis and necrosis assay.**

B16-OVA cells were pretreated with PBS, OVA or TPC@OVA NPs (30 μg mL^-1^), and 4 h later, the cells were irradiated with a 685 nm laser (0.6 W cm^-2^) for 5 min for light groups. After incubation at 37 ^o^C for 20 h, the cells were washed by PBS, then stained with Annexin-V-FITC and PI in binding buffer for 20 min. Finally, the apoptosis and necrosis assay were conducted by flow cytometer.

**2.8 HMGB1 release and CRT exposure.**

After incubating with TPC@OVA NPs (15 µg mL^-1^) for 4 h, the seeded B16-OVA cells on a confocal dish were washed about three times by PBS, followed by irradiating with laser for 5 min for light groups. After incubating another 3 h, the washed cells by PBS were infiltrated 4% paraformaldehyde for 20 min and Triton X-100 (0.1%) for 10 min (only HMGB1 release), followed by incubating with 1% BSA for 30 min. Afterwards, the primary antibody of CRT and HMGB1 was jointed and incubated at 4 ^o^C for 12 h, respectively. After removing the supernatant CRT and HMGB1 antibody, the cells were incubated with FITC labelled secondary antibody IgG at 4 ^o^C for 1 h. Finally, the washed cells by PBS were then stained cell nuclei with Hoechst 33258 for further CLSM imaging.

**2.9 Cellular ATP measurement.**

B16-OVA cells were seeded in 96-well plates and allowed to adhere for 24 h. Then the cells were cultured with PBS, OVA or TPC@OVA NPs (15 µg mL^-1^) at 37 ^o^C for 4 h, followed by irradiating with laser for 5 min for light groups. The ATP levels of cell supernatant were determined by Enhanced ATP Assay Kit according to the provided protocol.

**2.10 TNF-α, IFN-γ and IL-6 secretion.**

B16-OVA cells at a density of 5 × 10 ^3^ cells per well were seeded in 96-well plate and incubated overnight. Then B16-OVA cells were treated with PBS, OVA or TPC@OVA NPs (15 µg mL^-1^) at 37 ^o^C for 4 h, followed by irradiating with laser for 5 min for light groups. The supernatant of B16-OVA cells was collected and used as the culture medium for DC2.4 cells. And the incubation of DC2.4 cells was continued for another 12 h. The cells supernatant was collected to determine the secretion of immune inflammatory cytokines (TNF-α, IFN-γ and IL-6) by ELISA.

**2.11 DCs mature.**

B16-OVA cells at a density of 5 × 10 ^3^ cells per well were seeded in 96-well plate and incubated overnight. Then B16-OVA cells were treated with PBS, OVA or TPC@OVA NPs (15 µg mL^-1^) at 37 ^o^C for 4 h, followed by irradiating with laser for 5 min for light groups. The supernatant of B16-OVA cells was collected and used as the culture medium for DC2.4 cells. And the incubation of DC2.4 cells was continued for another 12 h. Then, the cells were washed by PBS, and then the cells were digested and centrifugated at 1800 r/min for 10 min. Next, PE-CD80, FITC-CD86 and APC-CD11c antibodies were added to stain the cells for 1 h. The cells were blocked with 5% BSA for 0.5 h, washed with PBS and centrifugated at 1800 r/min for 10 min. The supernatant was discarded, and 0.1 mL of PBS was added to resuspend the cells. The expression of CD80 and CD86 on DC2.4 cells was measured by flow cytometry.

**2.12 In vivo tumor suppression assay in mice.**

All animal experiments have been approved (No. 2024-00104) by the Academic Committee of Changchun Institute of Applied Chemistry, Chinese Academy of Sciences, and carried out according to the NIH guidelines for the care and use of laboratory animals (NIH publication No. 85-23 Rev. 1985). B16-OVA cells (1×10^6^ cells/100 μL) were injected into the right back of C57BL/6 mice to establish the primary tumor model. B16-OVA cells (5×10^5^ cells/100 μL) were injected into the back of C57BL/6 mice to establish the distant tumor model. When the primary tumor volumes reached about 80 mm^3^, the mice were randomly divided into 6 groups (*n* = 4). The body weight and tumor size were monitored every 2 d. After treatment, mice were sacrificed, and tumors and major organs were collected. After 14 days of treatment, mice were sacrificed, and the tumors and major organs (heart, liver, spleen, lung and kidney) were collected for H&E staining. The presence of TUNEL in tumor sections were investigated by immunofluorescence staining. The presence of Ki67 in tumor sections were investigated by immunohistochemical method.

**2.13 Analysis of immune response capacity in vivo.**

In order to analyze the immune response of TPC@OVA NPs, the spleens, lymph nodes and primary tumors in mice were surgically removed at 8 d post-treatment. The single-cell suspension from these tissues were prepared using the same protocol. Lymphocytes collected from the lymph nodes, tumor and spleen were incubated with fluorescent labeled antibodies CD8-APC, CD3-PE for 1 hour at 37°C, and the percentages of CD3^+^ CD8^+^ T cells were detected by flow cytometry. The collected lymphocytes from lymph nodes and spleen were incubated with fluorescent-labeled antibodies CD80-PE, CD86-FITC, CD11c-APC for 1 hour at 37°C, and the percentages of maturation DCs (CD80^+^ CD86^+^) were detected by flow cytometry. The collected cells from the tumor were incubated with fluorescent-labeled antibodies CD11b-APC and Gr-1-PE, and the percentages of MDSCs were detected by flow cytometry. Using ELISA kits to detect the levels of TNF-α, IFN-γ and IL-6 in mice serum was performed by a standard protocol. For immunofluorescence imaging, tumors were collected, fixed in 4% paraformaldehyde solution, and then embedded in paraffin, sliced. The presence of CRT, HMGB1, CD8a T cells and Tregs cells in tumor sections were investigated by immunofluorescence staining.

**Supplementary Figures**

**
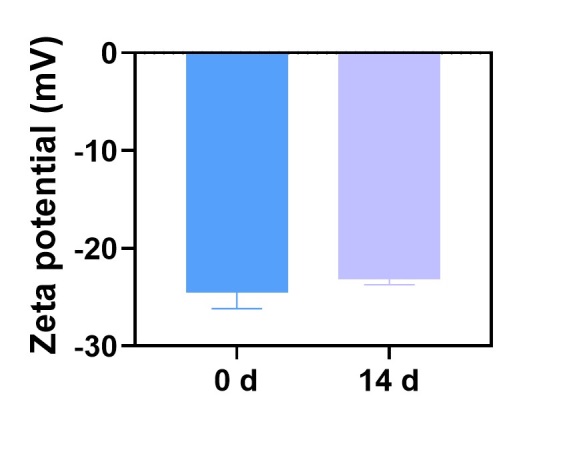
**

**Figure S1.** Zeta potential of freshly prepared TPC@OVA NPs stored for two weeks.

**
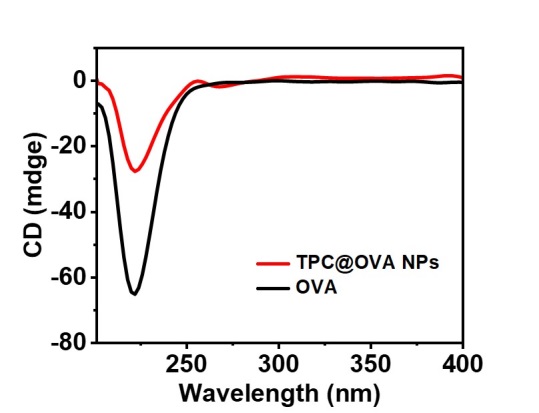
**

**Figure S2.** The CD spectra of TPC@OVA NPs and OVA.

**
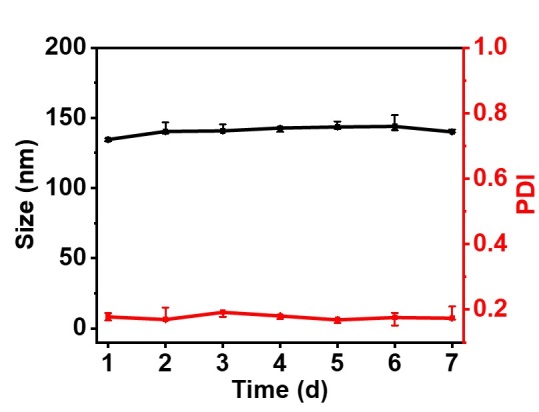
**

**Figure S3.** Size and PDI changes in deionized water for 7 days.


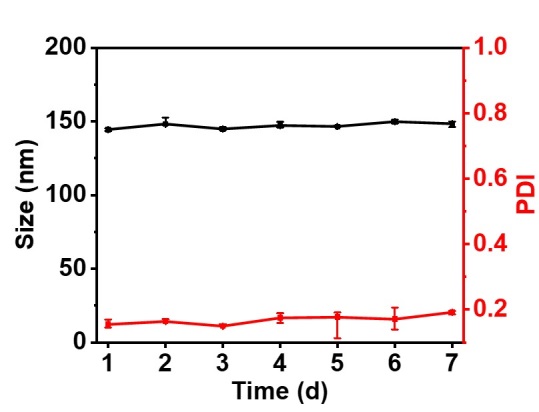


**Figure S4**. Size and PDI changes in 5% glucose solution.


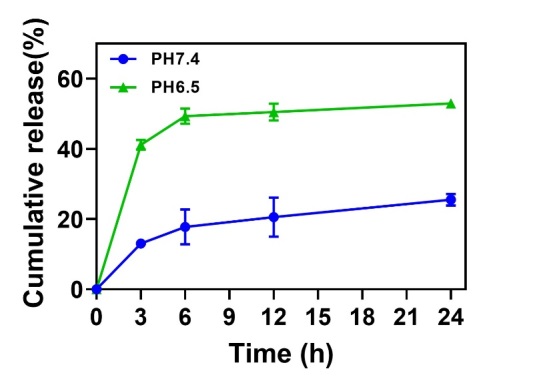


**Figure S5.** OVA release curves of TPC@OVA NPs in different pH solutions.


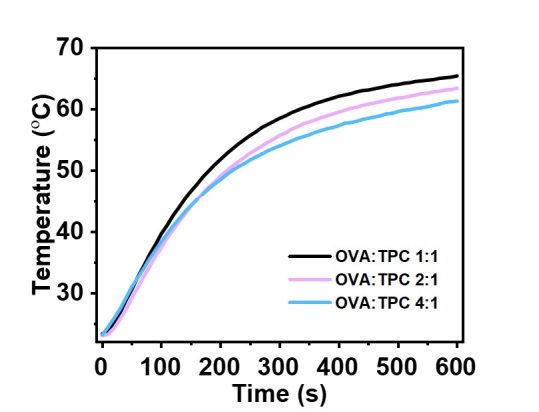


**Figure S6.** Photothermal property of TPC@OVA NPs with different proportions of OVA: TPC.


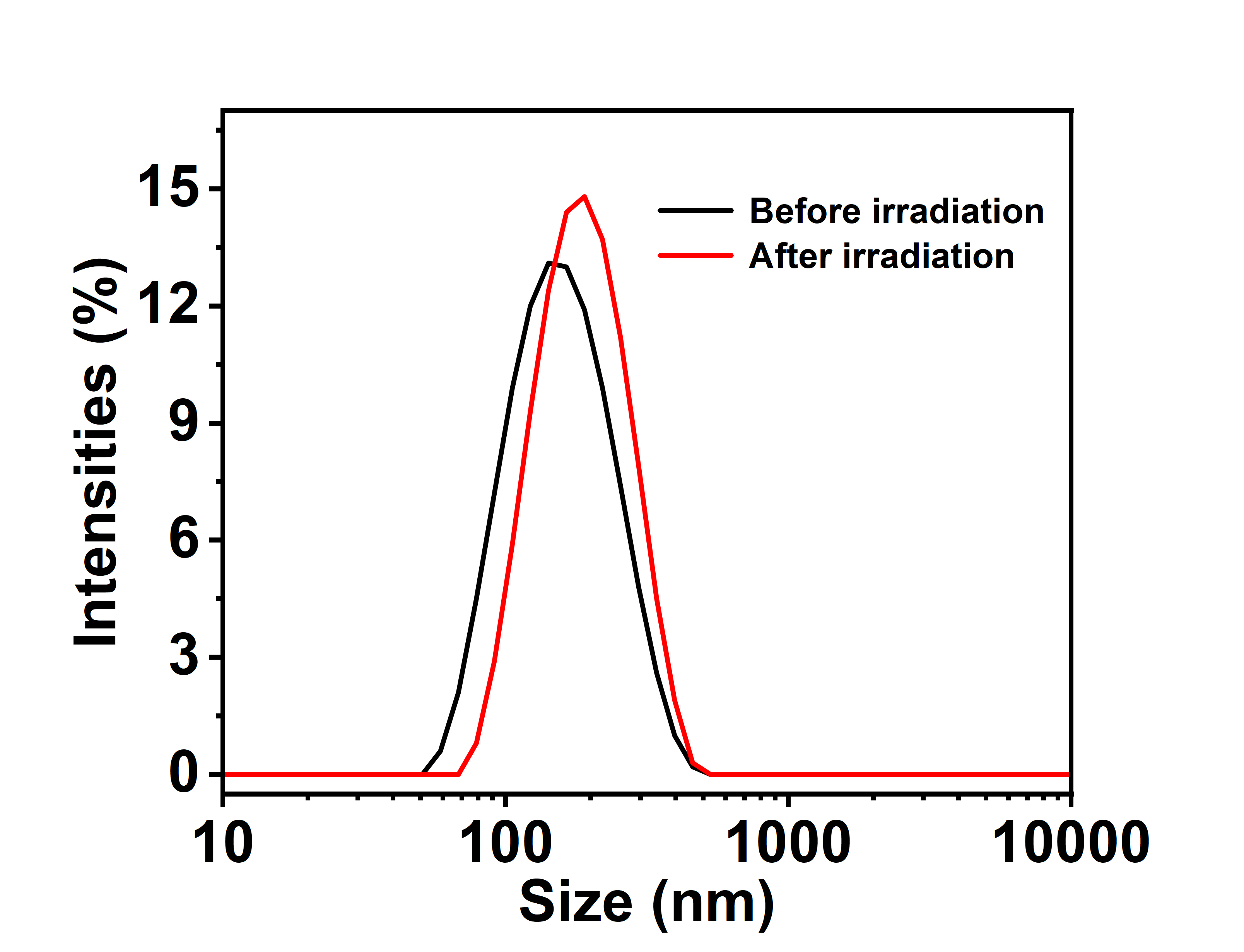


**Figure S7.** The size of TPC@OVA NPs before and after 5 cycles of irradiation.


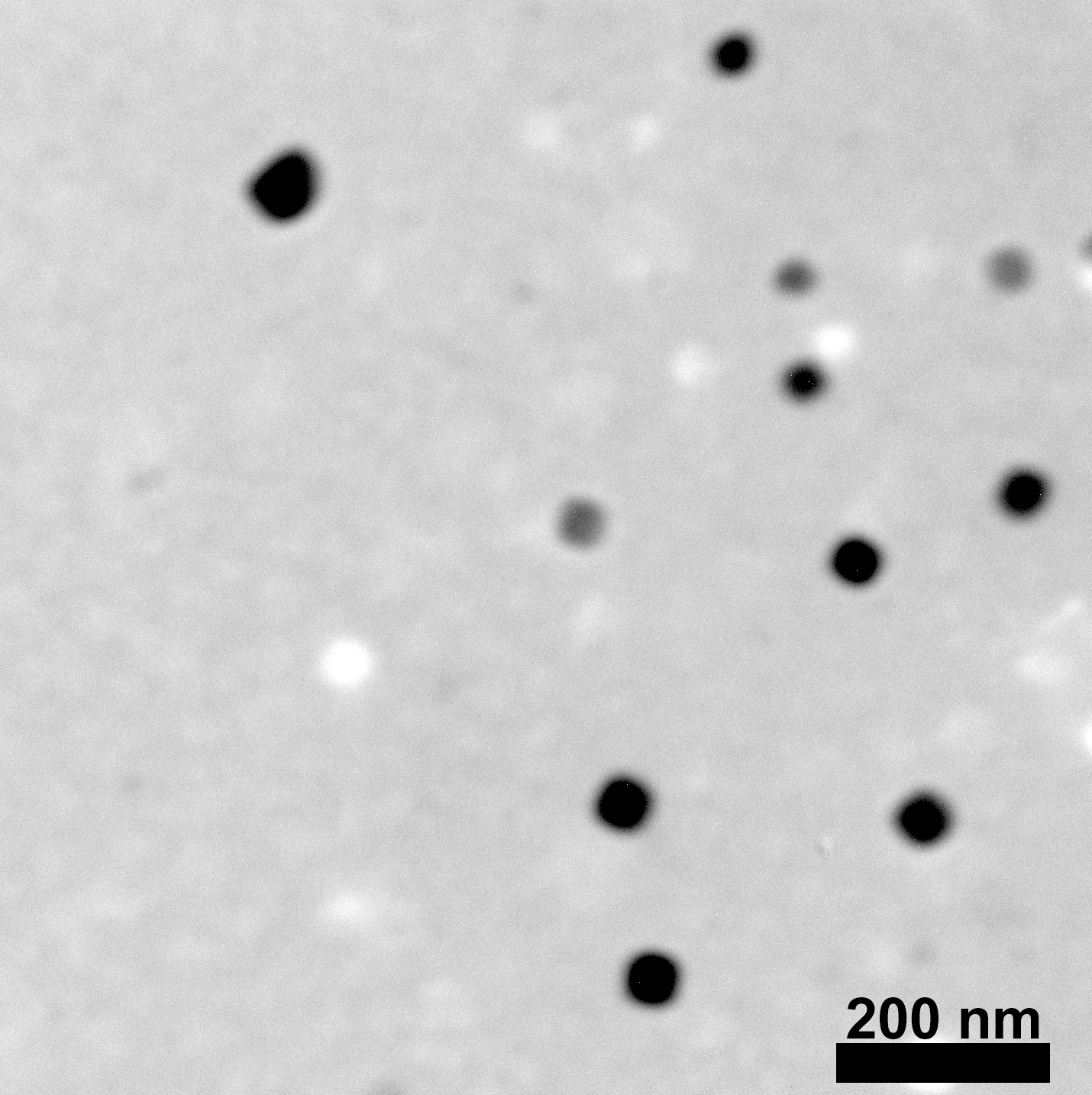


**Figure S8.** The TEM image of TPC@OVA NPs after 5 cycles of irradiation.


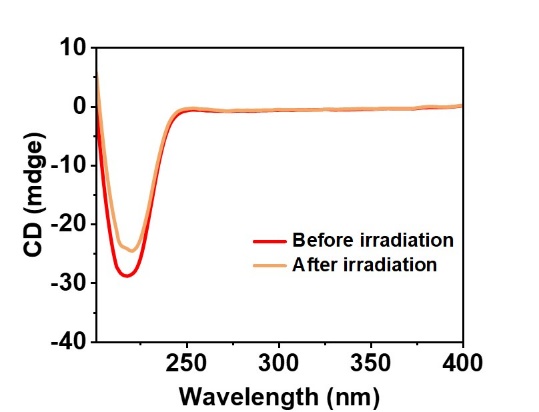


**Figure S9.** The CD spectra of TPC@OVA NPs before and after irradiation (685 nm, 600s).

**
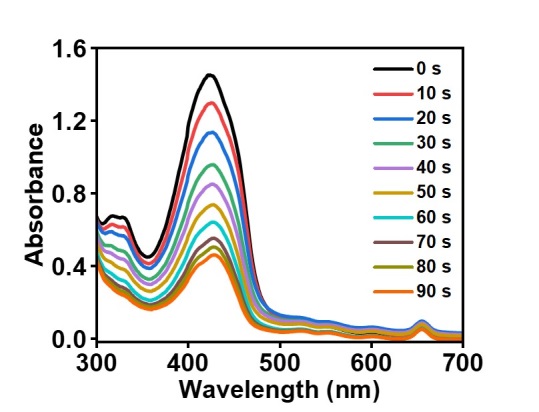
**

**Figure S10.** UV absorption spectra of DPBF at 417 nm with TPC@OVA NPs in solution after irradiation with 685 nm laser from 0 to 90 s.

**
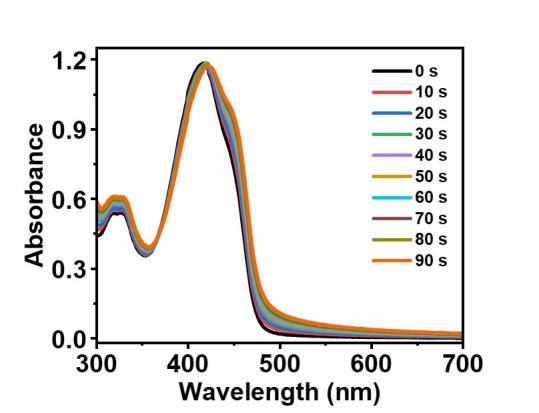
**

**Figure S11.** UV absorption spectra of DPBF at 417 nm in solution after irradiation with 685 nm laser from 0 to 90 s.


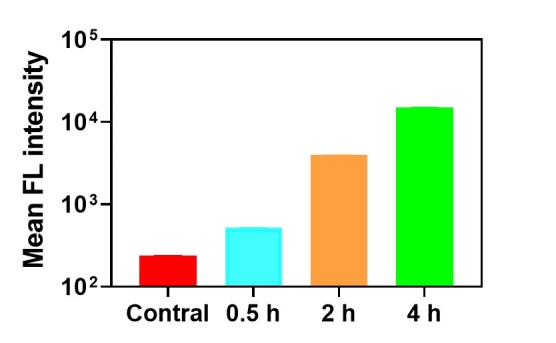


**Figure S12.** Mean ﬂuorescence intensity of B16-OVA cells after incubation with TPC@OVA NPs for 0.5, 2 and 4 h by FCM.


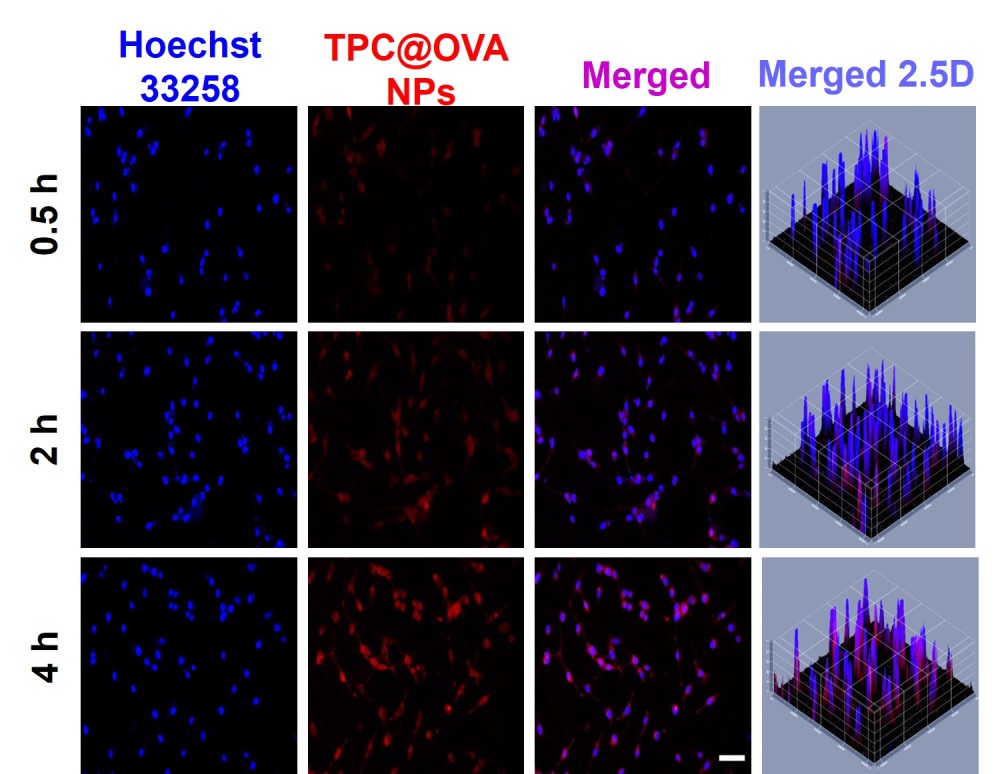


**Figure S13.** Fluorescent images of DC2.4 cells after incubation with TPC@OVA NPs for 0.5, 2 and 4 h. Scale bar, 50 μm.

**
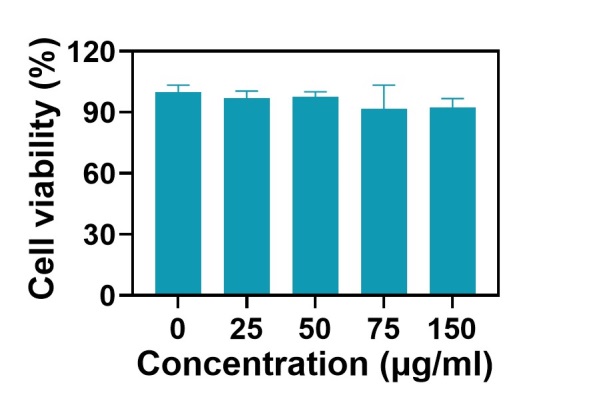
**

**Figure S14.** Cell viability of DC2.4 cells after incubation with TPC@OVA NPs of different concentrations.

**
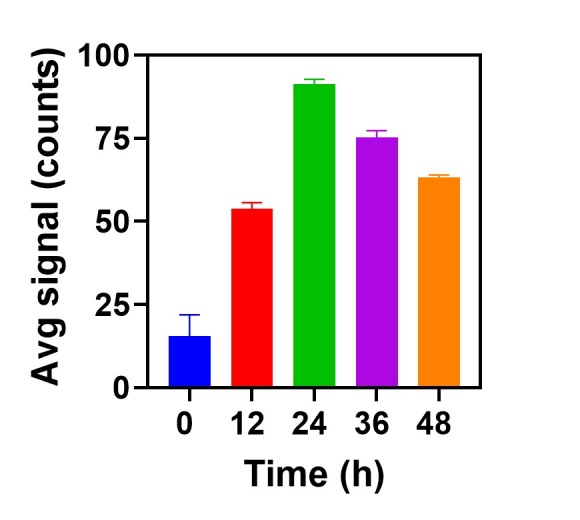
**

**Figure S15.** Mean fluorescence of tumors after intravenous injection of TPC@OVA NPs for different times.

**
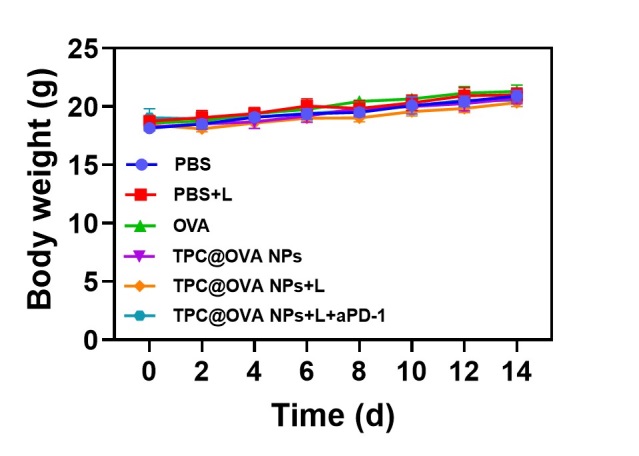
**

**Figure S16.** Changes of body weight of mice in each group during treatment.


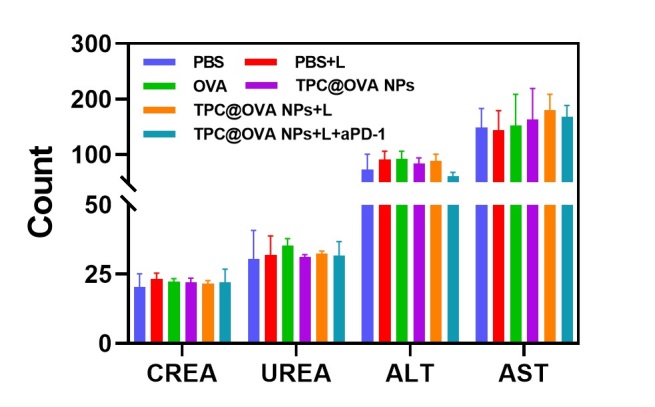


**Figure S17.** Blood biochemical indexes of mice after various treatments.

**
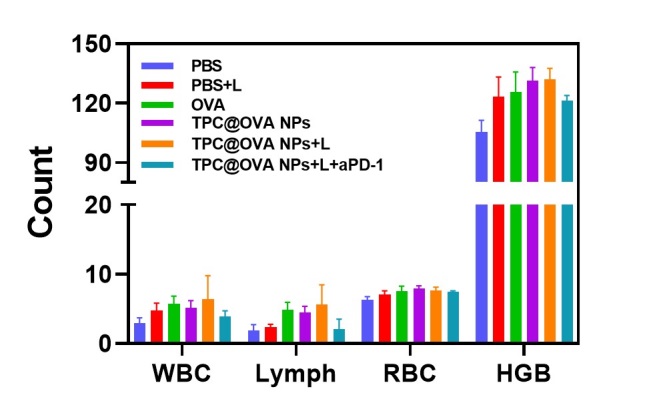
**

**Figure S18**. Routine blood analysis of mice after various treatments.


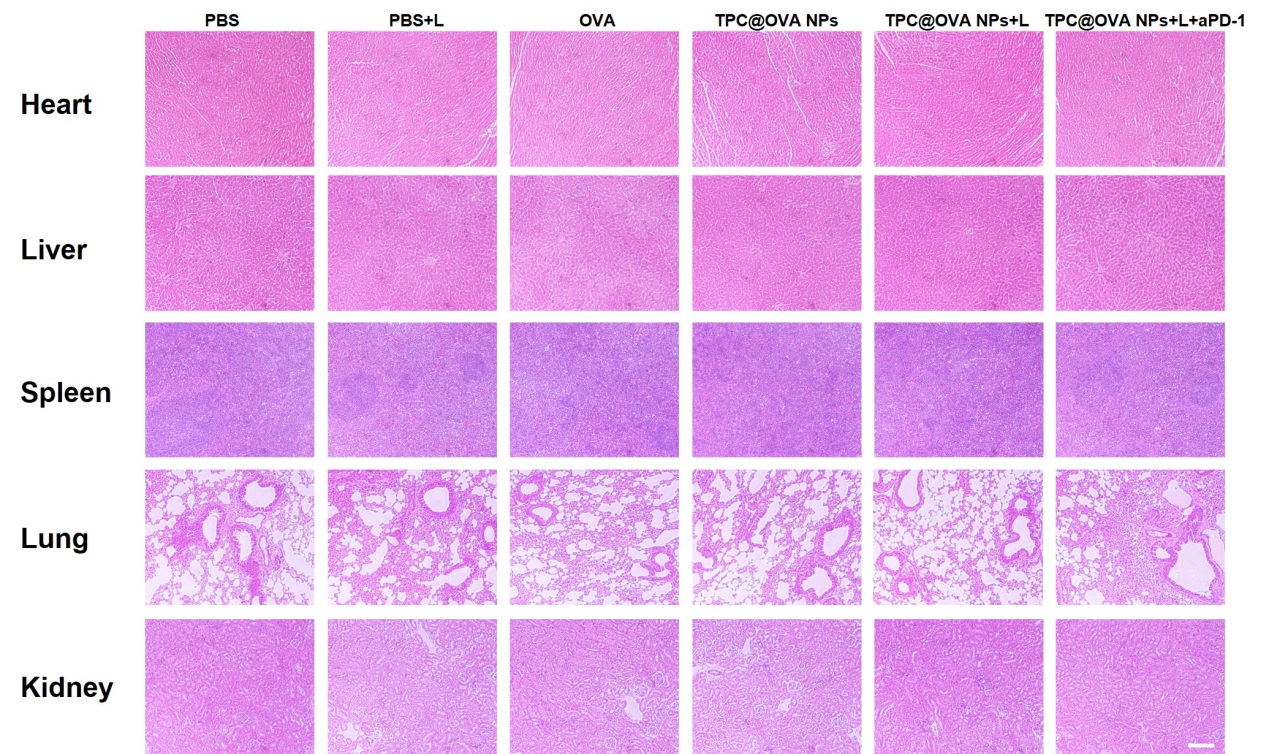


**Figure S19**. H&E staining images of the major organs (heart, liver, spleen, lung and kidney) from mice in each group. Scale bar: 100 μm.


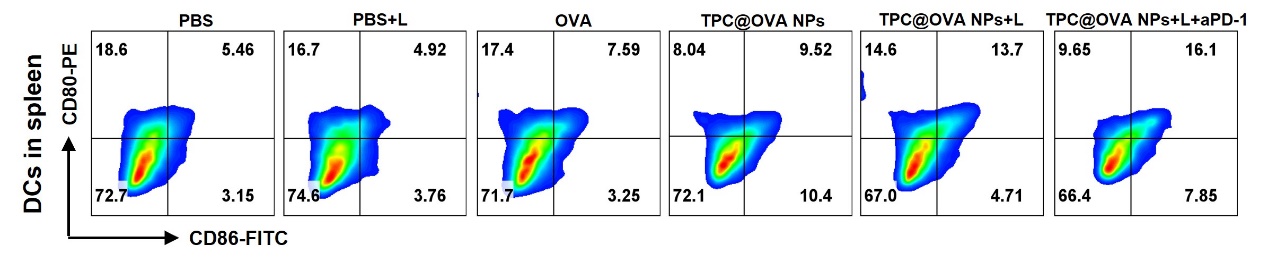


**Figure S20.** FCM analysis of maturated DCs in spleen in diﬀerent groups.


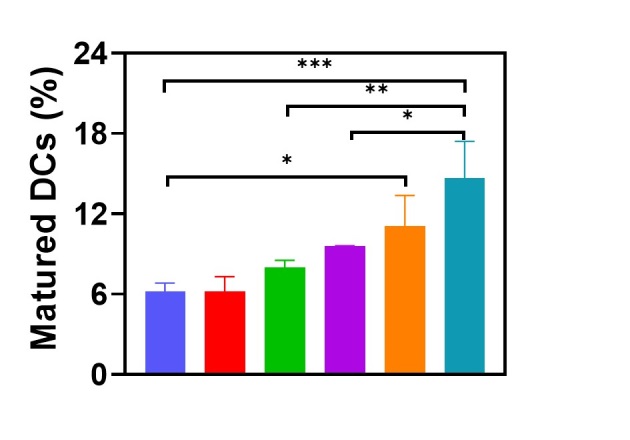


**Figure S21.** Corresponding quantitative result of maturated DCs in spleen in diﬀerent groups.

**
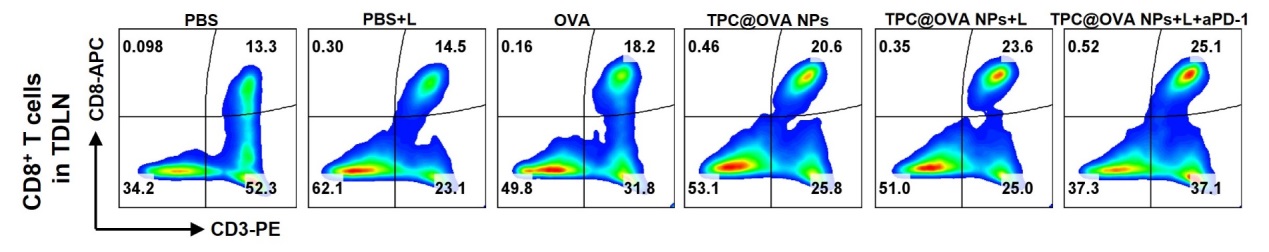
**

**Figure S22.** FCM analysis of CD8^+^ T cells in TDLN in diﬀerent groups.

**
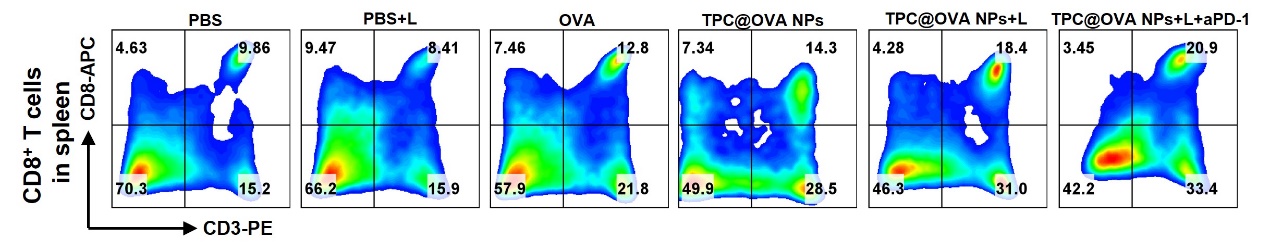
**

**Figure S23.** FCM analysis of CD8^+^ T cells in spleen in diﬀerent groups.

**Reference**

[1] X. Zheng, L. Wang, S. Liu, W. Zhang, F. Liu, Z. Xie, *Advanced Functional Materials* **2018**, *28*, 1706507.
